# Immune-Related Nine-MicroRNA Signature for Predicting the Prognosis of Gastric Cancer

**DOI:** 10.3389/fgene.2021.690598

**Published:** 2021-07-05

**Authors:** Jingxuan Xu, Jian Wen, Shuangquan Li, Xian Shen, Tao You, Yingpeng Huang, Chongyong Xu, Yaping Zhao

**Affiliations:** ^1^The Second Affiliated Hospital, Wenzhou Medical University, Wenzhou, China; ^2^The First Affiliated Hospital, Wenzhou Medical University, Wenzhou, China

**Keywords:** gastric cancer, overall survival, immune, microRNA, chemotherapy

## Abstract

Recent findings have demonstrated the superiority and utility of microRNAs (miRNAs) as new biomarkers for cancer diagnosis, therapy, and prognosis. In this study, to explore the prognostic value of immune-related miRNAs in gastric cancer (GC), we analyzed the miRNA-expression profiles of 389 patients with GC, using data deposited in The Cancer Genome Atlas database. Using a forward- and backward-variable selection and multivariate Cox regression analyses model, we identified a nine-miRNA signature (the “ImmiRSig,” consisting of *miR-125b-5p*, *miR-99a-3p*, *miR-145-3p*, *miR-328-3p*, *miR-133a-5p*, *miR-1292-5p*, *miR-675-3p*, *miR-92b-5p*, and *miR-942-3p*) in the training cohort that enabled the division of patients into high- and low-risk groups with significantly different survival rates. The ImmiRSig was successfully validated with an independent test cohort of 193 GC patients. Univariate and multivariate Cox regression analyses indicated that the ImmiRSig would serve as an independent prognostic factor after adjusting for other clinical covariates. Pending further prospective validation, the identified ImmiRSig appears to have significant clinical importance in terms of improving outcome predictions and guiding personalized treatment for patients with GC. Finally, significant associations between the ImmiRSig and the half-maximal inhibitory concentrations of chemotherapeutic agents were observed, suggesting that ImmiRSig may predict the clinical efficacy of chemotherapy.

## Introduction

Gastric cancer (GC) is the third major cause of cancer-related death and the fourth most common cancer worldwide; thus, GC is a key public health concern (Bray et al., 2018). Although early diagnosis and several different treatment strategies have improved the survival rate of patients with GC, the prognosis of patients with advanced GC remains unsatisfactory. The 5-year survival rate of advanced GC is approximately 20%, which is largely attributable to uncertain histopathological behaviors and metastatic characteristics in the early stages (Coburn et al., 2018). During GC development, tumor-induced immune suppression leads to imbalanced immune activity (Wang et al., 2017; Lazãr et al., 2018). GC immunotherapy, including immune-checkpoint therapy and cancer vaccines (which depend on the pertinent immunological mechanisms that moderate cancer development and occurrence), has improved in recent years (Martin et al., 2020). Nevertheless, immune heterogeneity, i.e., different immune cell banks and immune profiles, is associated with different responses to immunotherapy (Li J. et al., 2018; Ho and Tan, 2019). Recent progress brought about by omics research has enhanced our perception of the fundamental molecular mechanisms of GC and revealed a wide range of molecular heterogeneity between individuals, which emphasizes the importance of applying molecular biomarkers for clinical diagnosis and prognosis.

Differences in the survival rates of patients with GC are often caused by different genetic or molecular backgrounds. Gene-expression regulators play important roles in regulating immune responses and can mediate posttranscriptional gene modifications (Bradner et al., 2017). Therefore, research on tumor immunogenomics can broaden our understanding of precision medicine and tumor immune mechanisms.

Recently, a study focused on a newly discovered type of noncoding RNAs (ncRNAs), termed microRNAs (miRNAs), whose transcripts are approximately 20 nucleotides in length, has gained significant attention (Bavelloni et al., 2017). During the posttranscriptional regulation of gene expression, miRNAs play crucial functional roles by binding to 3′-untranslated regions of target messenger RNAs (mRNAs), thereby affecting their stabilities (Gebert and Macrae, 2019). Bioinformatic analysis of the predicted binding spectra of miRNAs showed that they directly influence the expression of most protein-coding genes (PCGs) in mammals (O’brien et al., 2018). Interestingly, miRNA genes are often located near cancer-related chromosomal regions and fragile locations in the genome (Georgakilas et al., 2014). Dysregulated biogenesis and expression of miRNAs are involved in tumor progression and initiation, therapy resistance, and metastasis formation, suggesting the existence of both tumor-suppressive and oncogenic miRNAs (Rupaimoole and Slack, 2017). Previous data have demonstrated that miRNAs may also exert cancer-causing or tumor-suppressing functions by affecting tumor immunogenicity or antitumor immune responses (Lin and Gregory, 2015; Hata and Kashima, 2016; Cortez et al., 2019; Verduci et al., 2019). Immune-related miRNAs have been associated with the occurrence, prognosis, and development of GC (Wu et al., 2014; Zhang et al., 2019b). Recent results have shown that some miRNAs belonging to the *miR-148* family (including *miR-152*, *miR-148B*, and *miR-148A*) can inhibit histocompatibility antigen, class I, G (HLA-G) expression, as well as the occurrence and development of GC (Zhai et al., 2014). Although the mechanisms of miRNAs in interferon-c-mediated HLA-G upregulation are unclear, an association has been documented between transforming growth factor-β-induced HLA-G expression and decreased *miR-152* expression in GC (Guan et al., 2015). miRNAs such as *miR-20a*, *miR-10b*, *miR-25*, *miR-93*, *miR-17-5p*, *miR-433*, and *miR-106b* can promote tumor growth; regulate the expression of genes encoding the major histocompatibility complex class I chain-related proteins A and B; increase tumor angiogenesis, cell proliferation, and invasion; and negatively interfere with natural killer cell-mediated cytotoxicity (Jasinski-Bergner et al., 2014; Xie et al., 2014).

In this study, we analyzed the expression profiles of immune-related genes and miRNAs in 389 patients with GC using data from The Cancer Genome Atlas (TCGA) project to systematically identify immune-related miRNAs and investigate their prognostic value. Using machine-learning methods, we identified and validated an immune-related nine-miRNA signature (ImmiRSig) that can help determine the prognosis of patients with GC in different cohorts.

## Materials and Methods

### GC Data Resource

The original clinical information and sequencing data used in the current study were derived from TCGA database^1^. We obtained miRNA-sequencing data of 491 samples, including 45 matched normal samples and 446 GC tissue samples and RNA-sequencing data of 375 GC and 32 normal tissue samples. The high-throughput sequencing-count expression-profiling data were used for our analysis.

We evaluated TCGA data from 420 patients with GC with miRNA-expression profiles and clinical follow-up information. All patients with GC were divided into a training cohort (to identify immune-related prognostic miRNAs) or a testing cohort (to validate the findings). The detailed clinical features of all GC cohorts are listed in Table 1.

**TABLE 1:** Clinical information of 389 TCGA GC patients.

Variables	Training cohort (*n* = 196)	Testing cohort (*n* = 193)	TCGA cohort (*n* = 389)
Clinical			
Age			
>65	107 (22.4)	99 (25.4)	205 (52.7)
≤65	87 (27.5)	93 (23.9)	181 (46.5)
Unknown	2 (0.5)	1 (0.3)	3 (0.8)
Gender			
Male	130 (33.4)	127 (32.6)	256 (65.8)
Female	66 (17.0)	66 (17.0)	133 (34.2)
Grade			
G1–2	72 (18.5)	73 (18.8)	145 (37.3)
G3	122 (31.4)	113 (29.0)	235 (60.4)
Unknown	2 (0.5)	7 (1.8)	9 (2.3)
Stage			
I	29 (7.5)	20 (5.1)	49 (12.6)
II	69 (17.7)	54 (13.9)	123 (31.6)
III	69 (17.7)	95 (24.4)	164 (42.2)
IV	20 (5.1)	17 (4.4)	37 (9.5)
Unknown	9 (2.3)	7 (1.8)	16 (4.1)
T			
T1	8 (2.1)	10 (2.6)	18 (4.6)
T2	48 (12.3)	36 (9.3)	85 (21.9)
T3	86 (22.1)	95 (24.4)	179 (46.0)
T4	50 (12.9)	51 (13.1)	102 (26.2)
Unknown	4 (1.0)	1 (0.3)	5 (1.3)
M			
M0	176 (45.2)	174 (44.7)	350 (90.0)
M1	11 (2.8)	14 (3.6)	25 (6.4)
Unknown	9 (2.3)	5 (1.3)	14 (3.6)
N			
N0	68 (17.5)	47 (12.1)	115 (29.6)
N1	51 (13.1)	57 (14.7)	108 (27.8)
N2	35 (9.0)	42 (10.8)	76 (19.5)
N3	36 (9.3)	42 (10.8)	79 (20.3)
Unknown	6 (1.5)	5 (1.3)	11 (2.8)
Disease type			
Adenomas and adenocarcinomas	176 (45.2)	179 (46.0)	355 (91.3)
Cystic, mucinous, and serous neoplasms	20 (5.1)	14 (3.6)	34 (8.7)

### Identification of Immune-Related miRNAs Associated With GC

Immune genes were downloaded from the ImmPort Shared Database^2^. Pearson correlations were used to assess association between miRNA-expression levels and immune-related genes. miRNAs that highly correlated with immune-related genes were defined as immune-related miRNAs. Differentially expressed immune-related miRNAs between the GC and normal samples that showed a | log_2_ fold-change| of >1 and a significant expression difference (*p* < 0.05) were identified as GC-associated immune-related miRNAs using the ‘‘edgeR’’ package of R software (R X86, version 4.0.3 for statistical computing, Vienna, Austria^3^).

### Development of Immune-Related miRNA Signature

Univariate Cox regression analyses were used to evaluate associations between overall survival (OS) and the expression levels of immune-related miRNAs in order to identify prognostic immune-related miRNAs. Forward- and backward-variable selection and multivariate Cox regression analyses were used to identify the optimal miRNA combination from the candidate prognostic immune-related miRNAs.

In the optimized model, the miRNAs and corresponding coefficients were presented, and the formula for calculating the prognostic index based on the optimal miRNA combination (designated as ImmiRSig) was obtained. For patients in both the testing and training cohorts, ImmiRSig was calculated based on the expression levels of immune-related miRNAs and their corresponding coefficients. In the training cohort, the patients were divided into high- and low-risk groups based on the median risk score. For high- and low-risk patients, survival analysis was performed using the “survival” package of R software, and area-under-the-curve (AUC) values were calculated based on receiver-operating characteristic (ROC) curves. Univariate and multivariate Cox regression analyses were performed to assess prognostic associations between risk scores and different variables, including sex, age, clinical stage, grade, and tumor–node–metastasis (TNM) stage.

### Correlation Analysis of Immune Cell Infiltration

The expression profiles of CD4^+^ T cells, B cells, dendritic cells, CD8^+^ T cells, neutrophils, and macrophages were downloaded from the Tumor Immune Estimation Resource (TIMER) database^4^. Using xCell^5^, TIMER, quanTIseq^6^, MCPcounter^7^, EPIC^8^, CIBERSORT-ABS, and CIBERSORT^9^ software, the Pearson correlation algorithm was used to calculate the correlations between risk scores and immune cells and to further determine whether a difference occurred in the expression profiles of immune cells in the high- and low-risk groups.

### Functional Enrichment Analysis

We performed a functional analysis to study the inherent biological effects of the nine-miRNA signature in GC. By searching miRDB^10^, miRTarBase^11^, and TargetScan^12^, we obtained the related target genes of miRNAs included in the ImmiRSig and selected overlapping miRNA target genes for further analysis. We calculated Pearson correlation coefficients to measure the coexpression associations between miRNAs and their target genes and formed a miRNA–target gene-interaction network. We analyzed miRNA-function enhancement and performed PCG-coexpression analysis based on Kyoto Encyclopedia of Genes and Genomes (KEGG) and Gene Ontology (GO) pathways to study the biological roles of the ImmiRSig in GC.

### Chemotherapeutic Response Prediction Analysis

People with advanced GC often need additional chemotherapy after radical surgery. Therefore, we used the “pRRophetic” package of R software to evaluate the responses to chemotherapy in high- and low-risk patients with GC. The responses were determined as half-maximal inhibitory concentrations (IC_50_s) for different anticancer drugs exhibited by different patients with GC.

## Results

### Identification of Immune-Related miRNAs Associated With GC Tumorigenesis and Outcomes

**FIGURE 1:**
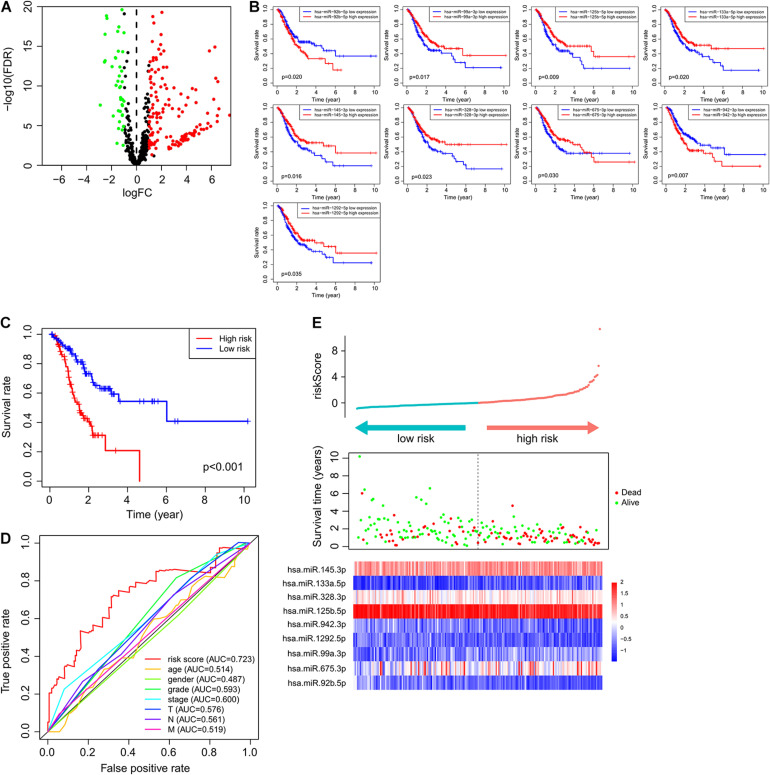
Immune-related miRNAs associated with GC tumorigenesis and outcomes. **(A)** MicroRNAs with differential expression. Green bubbles represented downregulated microRNAs, and red bubbles represented upregulated microRNAs. **(B)** Kaplan–Meier survival curve of nine prognostic microRNAs. **(C)** Kaplan–Meier survival curve of patients in high- and low-risk groups. **(D)** ROC curve of ImmiRSig, age, gender, grade, stage, and T, N, M in the training cohort. **(E)** Risk score distribution, survival status, and microRNA expression patterns for patients in high- and low-risk groups by the ImmiRSig in training cohort.

To identify immune-related miRNAs, we calculated Pearson correlation coefficients to measure the coexpression of miRNAs and immune-related genes. We identified 574 miRNAs coexpressed with immune genes as immune-related miRNAs. We subsequently compared the expression profiles of these immune-related miRNAs between normal and GC samples and identified 227 immune-related miRNAs that were differentially expressed (|log_2_ fold-change| > 1 and *p* < 0.05) and were possibly associated with GC tumorigenesis (Figure 1A). Of these miRNAs, 69 were downregulated and 158 were upregulated in GC samples, when compared with normal samples.

Overall survival was selected as the prognostic indicator. Only patients with a follow-up period longer than 30 days were included. Among the 420 GC samples, those from 389 patients (who had a follow-up period ranging from 30 to 3,720 days) were included in our analysis. Patients were randomly allocated to the training (*n* = 196) or testing (*n* = 193) cohorts using a 1:1 ratio and the “caret” package of R software.

In the training cohort, to identify immune-related miRNAs with prognostic value, univariate Cox proportional-hazards regression analysis was performed. Fifteen immune-related miRNAs were significantly associated with the OS rate of patients with GC (*p* < 0.05).

### Machine Learning-Based Derivation of the ImmiRSig for Predicting Survival

To identify a miRNA combination with optimal predictive performance, we performed an integrative analysis, based on forward- and backward-variable selection and multivariate Cox regression, for all 15 immune-related prognostic miRNAs. Nine of the 15 immune-related prognostic miRNAs formed an optimal miRNA combination (Table 2). Among the nine prognostic miRNAs, six miRNAs (*miR-125b-5p*, *miR-99a-3p*, *miR-145-3p*, *miR-328-3p*, *miR-133a-5p*, and *miR-1292-5p*) with a negative coefficient in the univariate-regression analysis were highlighted as possible tumor-suppressive factors due to the close associations between their high expression and prolonged patient survival. The remaining three miRNAs (*miR-675-3p*, *miR-92b-5p*, and *miR-942-3p*) tended to serve as risk factors, as their high expression was associated with poor survival prognosis (Figure 1B).

**TABLE 2:** Results of multivariate Cox regression analysis of nine prognostic immune-related miRNAs in the ImmiRSig.

miRNAs	Coefficient	HR (95% CI)	*p*-Value
miR-125b-5p	–0.4360	0.6466 (0.4499–0.9292)	0.0184
miR-1292-5p	–0.3057	0.7365 (0.5511–0.9844)	0.0387
miR-99a-3p	–0.1306	0.7174 (0.4732–0.9734)	0.0527
miR-145-3p	–0.2945	0.7449 (0.5863–0.9463)	0.0519
miR-328-3p	–0.2899	0.7483 (0.5898–0.9495)	0.0175
miR-133a-5p	–0.2744	0.7600 (0.5388–1.0721)	0.1179
miR-675-3p	0.1553	1.1679 (1.0473–1.3025)	0.0052
miR-92b-5p	0.3396	1.4044 (1.0610–1.8589)	0.0170
miR-942-3p	0.4053	1.4997 (1.0965–2.2569)	0.0158

To construct a clinically applicable predictive model, all nine immune-associated prognostic miRNAs were subjected to multivariate-Cox regression analysis to examine their relative contributions to predictive performance. The ImmiRSig was developed using a linear combination of the expression levels of all nine immune-associated prognostic miRNAs, weighted by the regression coefficients of multivariate Cox regression, as follows:

$$\begin{array}{c} ImmiRSig=\left(-0.4360\times \text{e}\mathrm{xpression}\mathrm{value}\mathrm{of}\mathrm{miR}-125b-5p\right)\\ \;+\left(-0.3057\times \mathrm{expression}\mathrm{value}\mathrm{of}miR-1292-5p\right)\\ \;+\left(-0.1306\times \mathrm{expression}\mathrm{value}\mathrm{of}miR-99a-3p\right)\\ \;+\left(-0.2945\times \mathrm{expression}\mathrm{value}\mathrm{of}miR-145-3p\right)\\ \;+\left(-0.2899\times \mathrm{expression}\mathrm{value}\mathrm{of}miR-328-3p\right)\\ \;+\left(-0.2744\times \mathrm{expression}\mathrm{value}\mathrm{of}miR-133a-5p\right)\\ \;+\left(-0.1553\times \mathrm{expression}\mathrm{value}\mathrm{of}miR-675-3p\right)\\ \;+\left(-0.3396\times \mathrm{expression}\mathrm{value}\mathrm{of}miR-92b-5p\right)\\ \;+\left(-0.4053\times \mathrm{expression}\mathrm{value}\mathrm{of}miR-942-3p\right) \end{array}$$

According to ImmiRSig, each patient’s risk score was computed in the training cohort. All patients in the training cohort were subdivided into high- (*n* = 98) and low-risk (*n* = 98) groups, based on the median risk score. As shown in Figure 1C, OS was significantly different between both risk groups (*p* = 1.2e−06, log-rank test). Patients in the low-risk group had significantly better OS periods than patients in the high-risk group.

Figure 1D shows the predictive performance of the ImmiRSig, estimated using time-dependent ROC curves for survival. The AUC for the ImmiRSig was 0.723, which was higher than that determined using other clinical features including age, sex, grade, the clinical stage, and the TNM stage. In the training cohort, we ranked the expression levels of the miRNAs in the ImmiRSig and analyzed their distributions (Figure 1E). For patients with low-risk scores, the expression levels of six protective miRNAs (*miR-125b-5p*, *miR-1292-5p*, *miR-99a-3p*, *miR-145-3p*, *miR-328-3p*, and *miR-133a-5p*) were upregulated, and the three risk-related miRNAs (*miR-675-3p*, *miR-92b-5p*, and *miR-942-3p*) were downregulated. Conversely, the expression levels of the prognostic miRNAs showed opposite patterns in high-risk patients.

### Independent Performance Validation of the ImmiRSig

**FIGURE 2:**
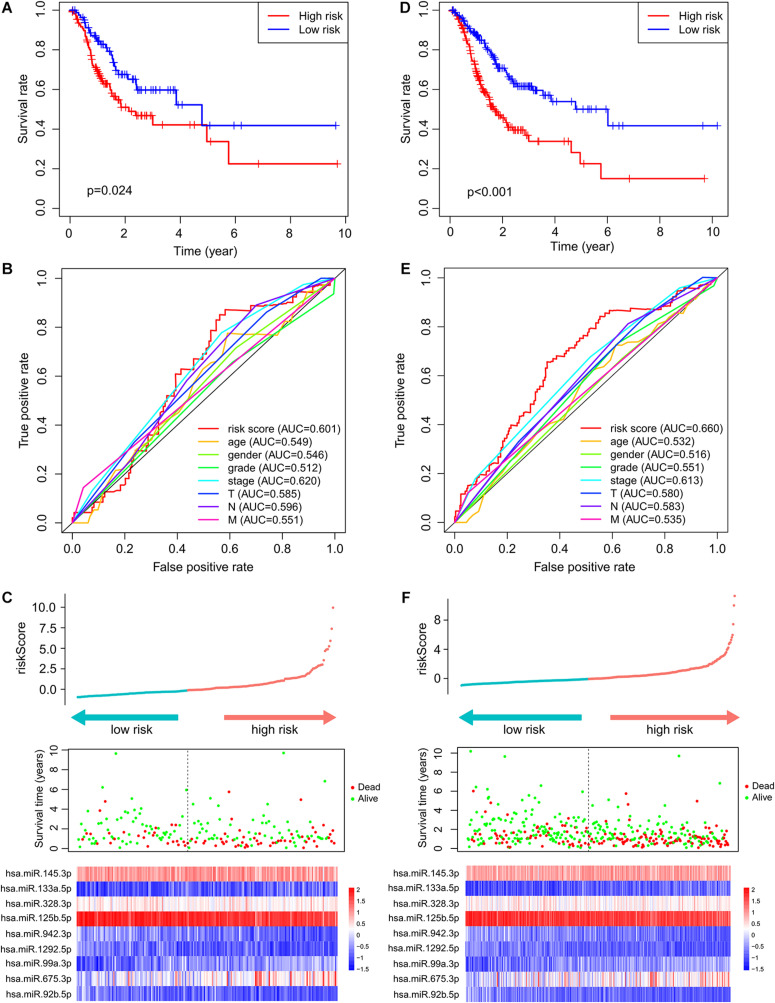
Independent validation of the ImmiRSig in different cohorts. The Kaplan–Meier survival curve of patients in high- and low-risk groups in the testing **(A)** and entire TCGA cohorts **(D)**. ROC curve of ImmiRSig, age, gender, grade, stage, and T, N, M in the testing **(B)** and entire TCGA cohorts **(E)**. Risk score distribution, survival status, and microRNA expression patterns for patients in high- and low-risk groups by the ImmiRSig in the testing **(C)** and entire TCGA cohort **(F)**.

To test the robustness of the ImmiRSig, data from the test and entire TCGA patient cohorts were studied to further verify the prognostic value of the ImmiRSig. Using the ImmiRSig, the prognostic risk score of patients in the test cohort was calculated based on the expression values of nine immune-related miRNAs for prognosis. By comparing the risk score of each patient in the test cohort with the cut-off value in the training cohort, the patients were classified as high- (*n* = 110) and low-risk (*n* = 83) patients. Kaplan–Meier survival curves revealed that the OS was significantly different between both risk groups (*p* = 2.436e−02, log-rank test), as shown in Figure 2A. Similarly, during the entire follow-up period, the survival rate of high-risk patients was lower than that of low-risk patients. Based on the ImmiRSig, ROC analysis was performed to assess the patient OS rate. In the test cohort, the ImmiRSig AUC was 0.601, which was comparable with that of the clinical stage (AUC = 0.62) and higher than other clinical features (Figure 2B). The survival status, risk score, and expression distribution of the ImmiRSig are shown in Figure 2C. Consistent with the training cohort, the expression levels of high-risk miRNAs in the low-risk group were lower than those in the high-risk group, whereas the expression levels of highly protective miRNAs in the low-risk group were higher than those in the high-risk group (Figure 2C).

The performance of the ImmiRSig in determining patient prognosis using data from the entire TCGA cohort was similar to the above results for the test cohort. The patients in the entire TCGA cohort were divided into the high- (*n* = 208) and low-risk (*n* = 181) groups. The OS rate of the low-risk group was significantly higher than that of the high-risk group. The ImmiRSig-based Kaplan–Meier survival curves differed significantly between both risk groups (*p* = 9.963e−07; Figure 2D). With the entire TCGA cohort, similar ROC analysis was performed and similar results were observed as described above. The AUC for ROC analysis of OS was 0.660 (Figure 2E). Figure 2F demonstrates the survival status, risk score, and distribution of long ncRNAs-expression levels in patients with GC in the entire TCGA cohort, with results similar to those observed in the training and testing cohorts.

### Independence of ImmiRSig From Molecular Features and Other Clinical Variables

**FIGURE 3:**
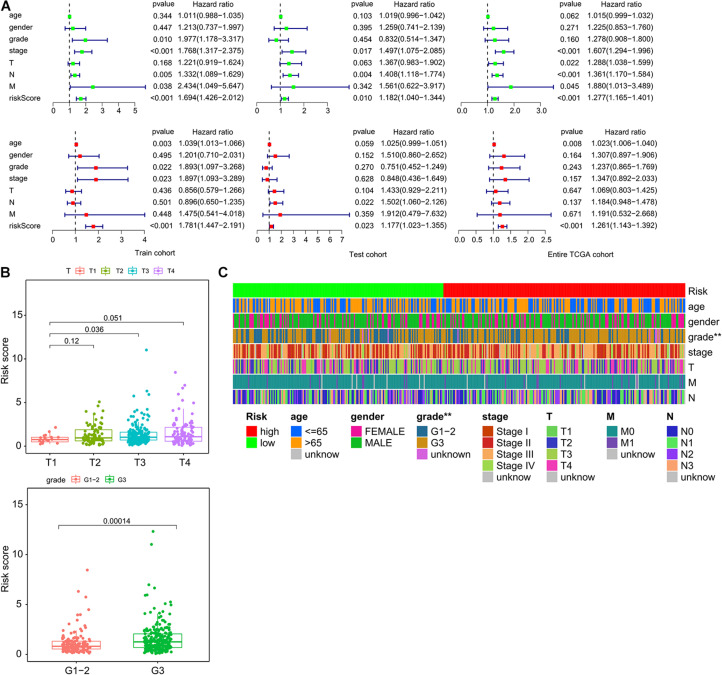
Independence of ImmiRSig from molecular features and other clinical variables. **(A)** Univariate and multivariate Cox analyses of ImmiRSig with other clinical variables. **(B)** Boxplot showing distribution of the ImmiRSig in patients with different stage and grade. **(C)** Distribution of molecular features and other clinical variables in the low-risk and high-risk groups.

Next, we investigated whether the prognostic value of the ImmiRSig was independent of other clinical variables. Multivariate-Cox regression analysis was performed using sex, age, clinical stage, grade, TNM, and ImmiRSig as covariates. Both the univariate and multivariate Cox analyses indicated that the ImmiRSig was significantly associated with OS after adjusting for age, sex, clinical stage, grade, and TNM stage (*p* < 0.05; Figure 3A). The ImmiRSig was confirmed as an independent predictor of good prognosis, irrespective of the clinical characteristics. The association between the ImmiRSig and each clinical characteristic (i.e., age, sex, grade, clinical stage, and TNM stage) was also evaluated. The risk score significantly correlated with the tumor grade and T stage (*p* = 0.036; Figure 3B). Additionally, the tumor grades differed significantly between the high- and low-risk groups (*p* = 0.00014; Figure 3C).

### Functional Analysis of the ImmiRSig

**FIGURE 4:**
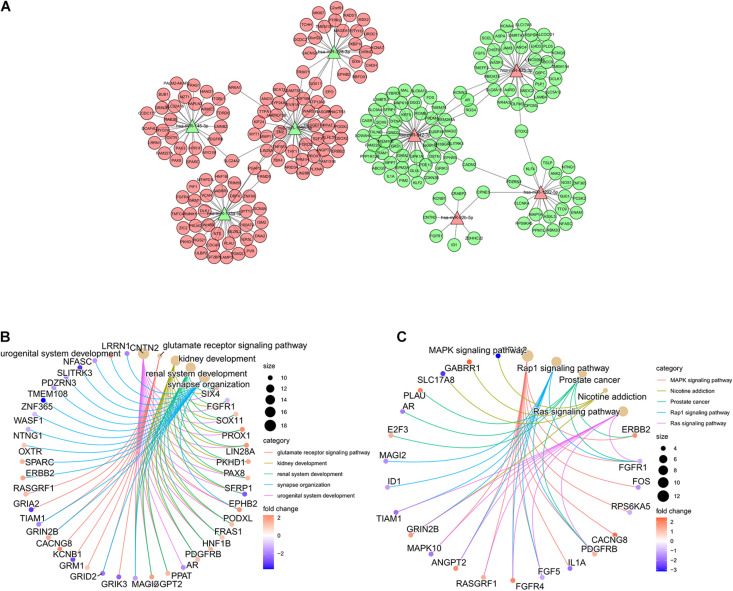
Functional analysis of the ImmiRSig. **(A)** Coexpression network of miRNA-target. **(B)** GO enrichment analysis. **(C)** KEGG enrichment analysis.

To better understand the functions of the miRNAs in the ImmiRSig, we performed functional analysis to reveal their potential biological effects, identified target genes for the miRNAs included in the ImmiRSig, and selected overlapping miRNA target genes for further analysis. We measured associations between miRNA and target gene coexpression and constructed a miRNA–target gene-interaction network including 198 connections between eight miRNAs and 225 mRNAs (Figure 4A). *MiR-99a-3p* did not show sufficient coexpression of target genes in a Venn diagram; thus, it was deleted from the interaction network. Of the remaining miRNAs, *miR-942-3p* and *miR-125b-5p* had the most connections with the candidate mRNA targets, indicating that they may be important or GC progression and metastasis. We subsequently performed KEGG and GO enrichment analyses for 225 coexpressed PCGs to understand the potential biological roles of the miRNAs. These studies revealed an overrepresentation of coexpressed PCGs with prognostic miRNAs involved in cancer-related biological processes and pathway terms, such as miRNAs in cancer, choline metabolism in cancer, transcriptional dysregulation in cancer, RAS signaling pathway, PI3K-Akt signaling pathway, mitogen-activated protein kinase (MAPK) signaling pathway, and developmental growth involved in morphogenesis (Figures 4B,C). These results demonstrate that the ImmiRSig may serve as a cancer-related biomarker set and that different prognostic miRNA-expression levels may affect the development, progression, and metastasis of GCs.

### The ImmiRSig Is Associated With the Immune Microenvironment

**FIGURE 5:**
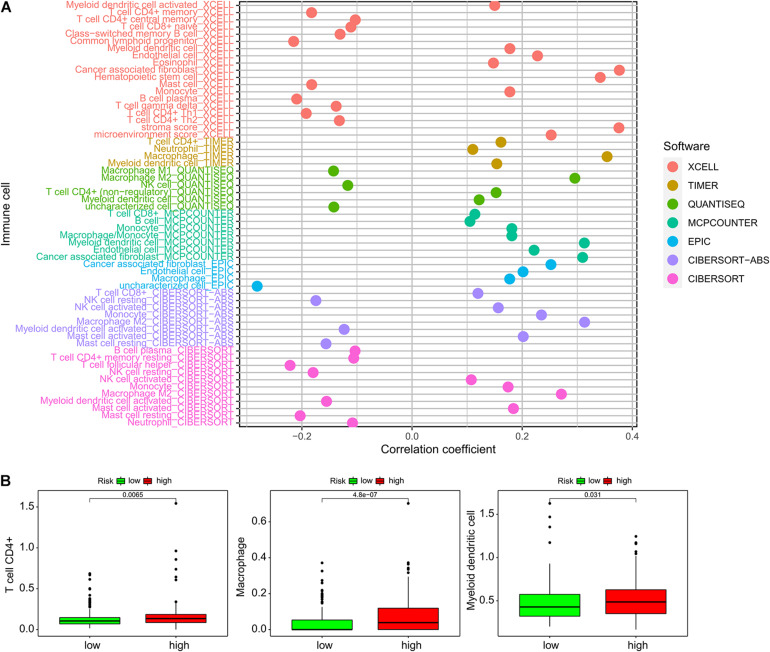
Association of ImmiRSig with chemotherapy response. **(A)** Paclitaxel IC_50_ between high- and low-risk patients with GC **(B)** sorafenib IC_50_ between high- and low-risk patients with GC.

We compared immune cell-infiltration levels for all 389 samples. The associations between each type of immune cell and the related risk scores were obtained (Figure 5A). CD4^+^ T cells, macrophages, and myeloid dendritic cells demonstrated significant differences in expression levels in the high- and low-risk groups. In the low-risk group, the levels of penetration of T cells, CD4^+^ macrophages, and myeloid dendritic cells were significantly lower than observed with the high-risk group (Figure 5B).

### Association Between ImmiRSig and Chemotherapy Response

**FIGURE 6:**
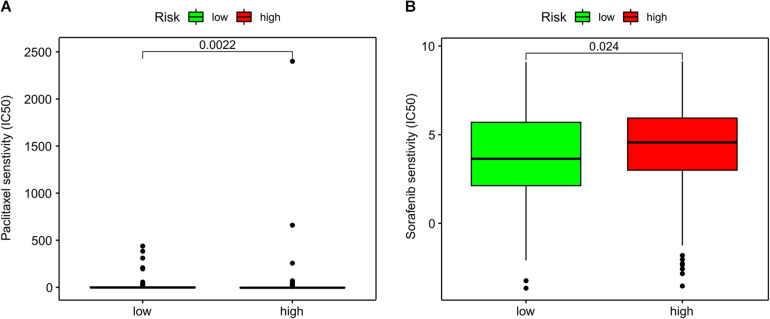
Association of the ImmiRSig with immune microenvironment. **(A)** The relationship between each immune cell and the ImmiRSig. **(B)** The infiltration levels of T cell CD4^+^, macrophages, and myeloid dendritic cells in the high- and low-risk group.

Subsequently, we investigated associations between the ImmiRSig and responses to chemotherapy. We found that sorafenib (*p* = 0.024) and paclitaxel (*p* = 0.0022) displayed significant differences in their estimated IC_50_ between high- and low-risk patients with GC. Notably, low-risk patients showed increased sensitivity to both chemotherapeutic agents (Figure 6).

## Discussion

Although aggressive and multimodal treatments (e.g., surgery, oxaliplatin-based chemotherapy, and immunotherapy) have improved the survival of patients with GC, the outcomes of patients with advanced GC remain unacceptable. At an advanced stage, patients with similar clinical risk factors for treatment have significantly different tumor responses and a different prognosis. Heterogeneous diseases are characterized by various pathological forms and molecularly heterogeneous changes, which (in addition to existing clinical risk factors) lead to an urgent need for determining other molecular prognostic indicators. GC is also a heterogeneous disease. Several molecular markers, such as mRNA or long ncRNAs, have been proposed over the past decade to support the prognosis of patients with GC (Xia et al., 2014; Sun et al., 2016; Cheng et al., 2018; Wang et al., 2020).

Some GC-specific biomarkers, including blood carcinoembryonic antigen CA 72-4 and carbohydrate antigen 19-9, have been studied through noninvasive testing. Newly identified prognostic biomarkers have primarily been used for the molecular analysis of tumors, including human epidermal growth factor receptor 2 (HER2) expression; the statuses of microsatellite instability/deficient mismatch repair (MMR) expression, programmed death-ligand 1 (PD-L1) expression, and Epstein–Barr virus infection; and detection of the neurotrophic tropomyosin receptor kinase fusion gene (Gravalos and Jimeno, 2008; Duffy et al., 2014; Shinozaki-Ushiku et al., 2015; Zhang et al., 2016; Cocco et al., 2018). The National Comprehensive Cancer Network Clinical Practice Guidelines in Oncology for GC have incorporated genetic biomarkers for the complex clinical management of patients with GC (NCCN, 2018), and the statuses of MMR protein expression, HER2 immunohistochemical staining, and PD-L1 expression have emerged as biomarkers. Based on the clinical-translation potential of miRNAs in controlling the expression of immune checkpoints, a previous study was conducted that revealed a correlation between the plasma levels of PD-1 and PD-L1, and the expression levels of immune-related miRNAs (Incorvaia et al., 2020). Some biomarkers are still being studied, including gene-expression profiles in tumorous tissues and circulating tumor cells in the peripheral blood, which have shown significant associations with clinical endpoints and were included in a randomized clinical study (Li T.t. et al., 2018).

Notably, validated biomarkers specific for GC are still limited and insufficient for supporting diagnostic and therapeutic requirements. Recent studies have shown that disturbances in miRNA-expression levels in various types of cancer have been widely observed, indicating the primary roles of miRNAs in cancer biology (Farazi et al., 2011; Shin et al., 2015).

In a previous study of a feature-selection procedure and classification model, five miRNAs (i.e., *miR-106b*, *miR-135b*, *miR-141*, *miR145*, and *miR-20a*) were selected as optimal biomarkers for diagnosing GC (Zhang et al., 2019a). Another study conducted using Cox regression and a robust likelihood-based survival model explored the prognostic signature of GC, and a three-miRNA signature (i.e., *miR-145-3p*, *miR-125b-5p*, and *miR99a-5p*) was established to help determine the prognosis of patients with GC (Zhang et al., 2018).

Our present findings highlighted immune-related miRNAs. Forward- and backward-variable selection and multivariate Cox regression analyses were performed to screen for survival-associated immune-related miRNAs. A survival-associated signature comprising the ImmiRSig, which included *miR-145-3p*, *miR-133a-5p*, *miR-328-3p*, *miR-125b-5p*, *miR-942-3p*, *miR-1292-5p*, *miR-99a-3p*, *miR-675-3p*, and *miR-92b-5p*, was constructed for patients with GC. A formula was established to obtain the risk score for evaluating the survival risks of patients.

Furthermore, among nine immune-related miRNAs, *miR-328-3p*, *miR-125b-5p*, *miR-942-3p*, *miR-1292-5p*, *miR-675-3p*, and *miR-92b-5p* were considered independent prognostic indicators of survival. These immune-related miRNAs may have distinct effects on the occurrence, progression, and metastasis of GC.

Among these six miRNAs, *miR-942-3p* showed the most connections with the mRNAs included in the regulatory network. We also explored the roles and functions of *miR-942-3p*, which was previously found to be upregulated in GC tissues (Chen et al., 2020), which is consistent with our current results. High levels of *miR-942-3p* expression increased the ImmiRSig score in patients with GC, suggesting that it may be an oncogenic factor in hepatocellular carcinoma (HCC) cells by targeting mannose-binding lectin 2 (Xu et al., 2019). *MiR-942-3p* promotes breast cancer progression by downregulating the expression of forkhead box protein A2 (Zhang J. et al., 2019). A new positive-feedback loop was identified between *miR-942-3p* and a transcriptional coactivator with a PDZ-binding motif, which regulated the biological function of bladder cancer cells and illustrated that these targets might have therapeutic potential as targets for prostate cancer treatment (Wang et al., 2021). The results of that study also suggest that *miR-942-3p* regulates the development of a variety of tumors. A few reports have discussed the association between *miR-675-3p* and cancer, and the literature shows that it is the target of several cancer-related mRNAs and interacts with multiple PCGs. These genes may participate in the competitive-endogenous RNA network in lung adenocarcinoma (Fan et al., 2020). Currently, the role of *miR-92b-5p* in GC remains undetermined; however, *miR-92b-5p* has been found to promote the proliferation and invasion of cervical cancer cells both *in vitro* and *in vivo*. *MiR-92b-5p* affects angiogenesis and the radiosensitivity of cervical cancer cells by targeting suppressor of cytokine signaling proteins (Wei et al., 2019). Platinum-based chemical resistance in metastatic cancer cells is associated with *MiR-92b*/glutathione *S*-transferase M3 (Ma et al., 2019). *MiR-1292-5p* showed a negative correlation with the risk-score formula. In a previous study, the *miR-1292-5p*–DEK axis was enhanced by the circular RNA circ0000039, thereby promoting GC progression (Gayosso-Gómez et al., 2014). These results differ from our current results and warrant further verification in a clinical trial. *MiR-328-3p* is also frequently described as a miRNA with a tumor-suppressor role in cancer. *miR-328-3p* can inhibit osteosarcoma cell proliferation and migration but accelerates programmed death by inhibiting matrix metalloproteinase-16 (Shi et al., 2019). *MiR-328-3p* acts as a tumor suppressor in HCC by inhibiting thioredoxin reductase 1 (TXNRD1), suggesting that it is a potential target for the clinical treatment of HCC (Li et al., 2019). In addition, *miR-328-3p* mimics are useful for treating colorectal cancer and can effectively predict tumor recurrence and treatment outcomes in patients with colorectal cancer (Wang et al., 2016; Ji et al., 2018). *MiR-125b-5p* is a commonly discussed immune-related miRNA that plays anticancer roles in several types of cancer. *MiR-125b-5p* was included in the three-miRNA-based risk score evaluation model for GC prognosis (Zhang et al., 2018). Li Y. et al. (2018) showed that by targeting KIAA1522, *miR-125b-5p* can inhibit tumor progression and regulate breast cancer progression. The *miR-125b-5p*–hexokinase 2 pathway has been reported to inhibit the progression of squamous cell carcinoma by regulating laryngeal squamous cell carcinoma glycolysis (Hui et al., 2018). Additionally, by inhibiting TXNRD1, *miR-125b-5p* was found to act as a tumor suppressor in HCC, making it a potential target in the clinical treatment of HCC (Hua et al., 2019).

The prognostic efficacy of the ImmiRSig was well validated in this study. Survival analysis was performed for both the training and test groups. Kaplan–Meier-survival curves indicated that the survival probability of patients in the high- and low-risk groups could be predicted with statistical significance.

The results of our GO and KEGG analyses indicated that cancer-related pathways, including the MAPK, Rap1, RAS, and other signaling pathways, were the most enriched pathways. The MAPK pathway leads to malignant phenotypes such as autonomic cell proliferation, which is often activated in human cancers (Sumimoto et al., 2006). Rap1 serves several roles in the invasion and metastasis of different cancers by interacting with other proteins (Zhang et al., 2017). Additionally, abnormal activation of the RAS protein is the main oncogenic factor that controls the function of the main signaling pathways involved in the occurrence and development of human malignant tumors (Zenonos and Kyprianou, 2013; Khan et al., 2019).

Intravenous chemotherapy is a very common method for treating patients with GC. Previous data showed that the correct administration time, dose intensity, and drug-effect ratio of paclitaxel combined with other chemotherapeutic drugs can safely be used as first-line treatment for patients with advanced GC, with high activity and a curative effect (Bruera et al., 2018). Our current findings confirmed that low-risk patients were more sensitive to paclitaxel and sorafenib than high-risk patients. Because of high immunosuppression and low immunoreactivity in the tumor microenvironment, these differences promote tumor growth, progression, invasion, and metastasis, which can contribute to a poor prognosis in high-risk patients. These differences suggest that high-risk patients may have lower benefits from immunotherapy and chemotherapy than low-risk patients.

The immune cell-infiltration levels were estimated using various software programs, and the results were compared across 389 patients. We identified significant differences in the infiltration of CD4^+^ T cells, macrophages, and myeloid dendritic cells in the high- and low-risk groups, suggesting a potential cellular mechanism. The limitation of this study is that, although the functional implication of the ImmiRSig was inferred by *in silico* functional analysis, careful functional characterization of the miRNAs should be performed in biological experiments, such as with RNA interference or *in vitro* cell-based assays. A well-designed and controlled clinical study should be conducted to verify the prognostic efficacy of the ImmiRSig.

## Conclusion

In conclusion, our results established the ImmiRSig for predicting the OS of patients with GC. MiRNAs were identified sequentially using univariate Cox analysis, multivariate-regression analysis, and forward- and backward-variable selection. The ImmiRSig score can be calculated using the expression levels of immune-related miRNAs and their respective coefficients. Furthermore, the identified immune-related miRNAs may be promising candidates as therapeutic targets.

## Data Availability Statement

The datasets presented in this study can be found in online repositories. The names of the repository/repositories and accession number(s) can be found in the article/supplementary material.

## Ethics Statement

The studies involving human participants were reviewed and approved by The Second Affiliated Hospital, Wenzhou Medical University, Wenzhou, China. The patients/participants provided their written informed consent to participate in this study.

## Author Contributions

JX and JW contributed to the study concepts and study design. JX and SL contributed to data acquisition and reconstructions. JX, XS, and TY contributed to the data analyses and interpretation. YH contributed to the statistical analysis. CX and YZ contributed to the manuscript preparation and manuscript editing and reviewing. All authors read and approved the final manuscript, contributed to the article, and approved the submitted version.

## Conflict of Interest

The authors declare that the research was conducted in the absence of any commercial or financial relationships that could be construed as a potential conflict of interest.

## Footnotes

1 https://cancergenome.nih.gov/

2 https://immport.org/shared/

3 www.R-project.org

4 https://cistrome.shinyapps.io/timer/

5 https://xcell.ucsf.edu/

6 https://icbi.i-med.ac.at/software/quantiseq/doc/

7 https://cit.ligue-cancer.net/mcp-counter/

8 https://gfellerlab.shinyapps.io/EPIC_1-1/

9 https://cibersort.stanford.edu/

10 http://mirdb.org/

11 https://bio.tools/mirtarbase

12 http://www.targetscan.org/vert_72/
